# Gastroprotective effect of the hydroethanolic extract of geopropolis produced by *Melipona subnitida* (Meliponinae, Apidae) in Wistar rats

**DOI:** 10.17221/98/2024-VETMED

**Published:** 2025-04-28

**Authors:** Jael Soares Batista, Tiago da Silva Teofilo, Helio Noberto de Araujo Junior, Natanael Silva Felix, Kaliane Alessandra Rodrigues de Paiva, Tabatta Arrivabene Neves, Lucas dos Santos Reboucas, Gerard Vicente Dantas de Medeiros, Kizzy Millenn de Freitas Mendonca Costa, Francisco Antonio Felix Xavier Junior, Giuseppina Negri, Antonio Salatino, Carmem Eusebia Palacios Jara

**Affiliations:** ^1^Department of Agrarian Sciences, Federal Rural University of the Semi-Arid Region (UFERSA), Mossoro, RN, Brazil; ^2^Center for Education, Science and Technology of the Inhamuns Region, State University of Ceará, Taua, CE, Brazil; ^3^Department of Botany, University of Sao Paulo, Sao Paulo, SP, Brazil

**Keywords:** gastric ulcers, gastroprotective, geopropolis, phenolic compounds, stingless bees

## Abstract

The jandaíra bee (*Melipona subnitida*) is a species native to northeastern Brazil that produces geopropolis, a mixture of clay and propolis, used in folk medicine. Geopropolis has traditionally been used in folk medicine because of its potential therapeutic properties, including antimicrobial, anti-inflammatory, and wound-healing effects. Recent studies have highlighted the rich composition of bioactive compounds such as flavonoids and phenolic acids, contributing to their pharmacological potential. Despite these findings, the gastroprotective properties of geopropolis and the underlying mechanisms remain underexplored and warrant further investigation. Therefore, we aimed to evaluate the gastroprotective effects of a hydroethanolic extract of geopropolis (HEG) produced by *M. subnitida* in Wistar rats, focusing on its antioxidant activity and the role of its bioactive compounds in preventing gastric mucosal damage. The gastroprotective potential was evaluated in Wistar rats pre-treated with HEG (250, 500, and 1 000 mg/kg, orally) for seven days and subjected to acute gastric lesions with ethanol (0.2 ml/animal, orally). One group of rats that received only distilled water served as the negative control, whereas the other group that received only ethanol served as the positive control. The stomachs were evaluated to determine the following parameters: evidence of macroscopic and histological changes, volume of mucus-containing mucin, stomach pH, and index of ulcerative lesions. Kolmogorov-Smirnov and Levene tests were performed, followed by the Tukey test, with values considered significant at *P < *0.05. HEG reduced the severity of the ulcerative lesions at all doses tested. Additionally, there was no significant difference in the pH values of gastric secretions, mucus volume, and mucin content in the stomachs of animals pretreated with HEG compared to the negative control group. These results indicate that HEG has gastroprotective activity, which may be related to the presence of phenolic compounds and its high antioxidant activity.

Approximately 250 native bee species, belonging to the Meliponini tribe (Hymenoptera: Apidae), have been identified in Brazil. They are commonly known as indigenous stingless bees or meliponines. These bees are responsible for pollinating various plant species and are, therefore, considered extremely important in maintaining natural and agricultural ecosystems (Sauthier et al. 2023). Meliponiculture is the breeding of bees of the Meliponini tribe. In Brazil, this activity has been traditionally practiced by indigenous communities and family farmers. Bee products, such as honey, wax, propolis, royal jelly, and pollen, produced by meliponids, are used as food and medicines to treat various diseases or are sold in informal markets (Souza et al. 2004; Lavinas et al. 2019).

Some Meliponidae species produce geopropolis, which consists of a mixture of resins, exudates from various plant sources, digestive enzymes, and clay. Geopropolis serves as a raw material for building hive structures and protecting against climatic factors and natural enemies (da Cunha et al. 2013; Ferreira et al. 2020). The chemical composition of the geopropolis is influenced by the species of bees that produce it, the climate, local flora, and soil characteristics of the region (Ferreira et al. 2020).

*Melipona subnitida*, also known as the jandaíra bee, is native to northeastern Brazil. The bees are docile and easy to manage and raise. They are adapted to extreme temperature conditions and long periods of drought occurring in the semi-arid climate of the northeast region (Fontoura et al. 2020). Although geopropolis produced by this species has been widely used in folk medicine for decades, information on its chemical composition, physical characteristics, and therapeutic properties is lacking.

Recent scientific research has shown promising results regarding the medicinal properties and beneficial effects of geopropolis. Research has shown that geopropolis contains several phenolic compounds, including flavonoids, and has anti-inflammatory and healing properties (Fontoura et al. 2020), high antioxidant capacity (Ferreira et al. 2023), and is photoprotective (Batista et al. 2021), antimicrobial (Santos et al. 2023), and hepatoprotective (Paiva et al. 2023).

Gastric ulcer is a common digestive disorder in humans with a high prevalence worldwide. Thus, there is a growing interest in studies related to the gastroprotective effects of natural products, to develop new drugs to expand the range of effective medicines with fewer side effects, low cost, and easy access (Oliveira et al. 2022). However, there is still no scientific evidence of the efficacy of geopropolis in preventing and treating gastrointestinal tract diseases. In this context, this study aimed to evaluate the gastroprotective effects of HEG produced by *M. subnitida* in the semiarid northeastern region of Brazil.

## MATERIAL AND METHODS

### Geopropolis collection and extract preparation

The geopropolis sample was directly collected from apiaries situated in the city of Mossoró, Rio Grande do Norte, Brazil (37º20’39” W 05º11’15” S). The region features a warm semi-arid tropical climate with an annual average temperature of 27 ºC, relative humidity of 50%, and an average annual rainfall of 500 mm.

### Analysis of chemical composition and antioxidant activity

Ten grams of crude geopropolis, previously ground in a ball mill, was extracted in a Soxhlet apparatus using 150 ml of ethanol for 6 hours. After extraction, insoluble residues were separated from the ethanolic solution by cold filtration through filter paper. The solvent was removed using a rotary evaporator at 40 ºC, resulting in an HEG extract.

The HPLC-DAD-ESI-MS/MS method was employed to determine the classes of bioactive compounds present in the HEG. Compounds were identified by mass spectrometry (MS), and retention times were compared with reference standards following the method described by Ferreira et al. (2017).

An *in vitro* photocolorimetric method described by Mensor et al. (2001) was used to evaluate the antioxidant activity of HEG. This method assesses the reduction of the DPPH (2,2-diphenyl-1-picrylhydrazyl) free radicals by antioxidants present in the extract. The absorbance value obtained using a spectrophotometre indicates the efficiency of free radical removal.

### Experimental design and gastric ulcer induction

Fifty adult male Wistar rats were used to assess the gastroprotective activity. The animals were housed in polypropylene boxes lined with wood shavings, maintaining a controlled temperature of 21 °C to 23 °C and a photoperiod of 12 h light and 12 h dark. They were provided with food (23% crude protein, 4% total lipids, 5% fibre, and 12% minerals) and water *ad libitum*. The experimental protocol was approved by the Animal Ethics Committee (CEUA) of the State University of Rio Grande do Norte (UERN) under the Protocol No. 006/15).

The animals were randomly divided into five experimental groups, each consisting of 10 animals, and they were administered by oral gavage as follows: Group G1 was treated with distilled water (negative control), and Group G2 was treated with ethanol puriss. p.a., absolute, ≥ 99.8% (positive control); and Groups G3, G4, and G5, pre-treated with HEG at doses of 250 mg, 500 mg, and 1 000 mg, respectively. One hour after the last administration of HEG, rats in Groups G3, G4, and G5 were subjected to acute gastric lesions induced by ethanol (0.2 ml/animal; oral gavage).

### Evaluation of mucus volume, macroscopic changes in the stomach, and ulcerative lesion index

The animals were euthanised by intraperitoneal administration of 400 mg/kg sodium thiopental 60 min after ethanol administration. The stomach was surgically removed, and the cardia and pyloric regions were clamped to prevent the loss of gastric contents. The stomach of each animal was incised at the greater curvature, emptied, and rinsed under gently running cold tap water to remove food debris. The mucus was removed from the mucosal surface using an automatic pipette to determine the secretion volume according to the adapted methodology of Taylor et al. (2004).

Macroscopic evaluation of the stomach involved visual observation of the organ’s mucosa and investigation of colouration, oedema, haemorrhage, and the presence of ulcerations. The intensities of these alterations were classified as absent (0), mild (+), moderate (++), or intense (+++). Photographs of the stomach mucosa were taken, and the images obtained were analysed using ImageJ software, allowing for the assessment of ulcer size and determination of the ulcerative lesion index (ULI), according to Sales et al. (2019).

### Evaluation of stomach pH

Immediately after opening the stomach, gastric mucus was aspirated by suction with a graduated pipette, the mucus volume was measured, and the results were expressed in millilitres. Next, a mucus sample from each animal was centrifuged (1 500 × *g*, 25 °C, 10 min), transferred to a glass tube, and 4 ml of distilled water was added. A portable, pH-sensitive, calibrated electrode (pH 4.0, 7.0) was inserted into the tubes to measure the mucus pH.

### Histopathological and histochemical assessment

Histological examination observed tissue lesions in fragments collected from the fundus, body, and pylorus. The samples were fixed in 10% formalin, embedded in paraffin, and histologically processed. Sections were cut at a thickness of 5 microns using a microtome and stained with haematoxylin and eosin. A single examiner, blinded to the experimental groups, conducted the histological analysis, described the observed alterations, and classified the lesions as absent (0), mild (+), moderate (++), or intense (+++).

Additionally, stomach fragments were subjected to histochemical staining with periodic acid-Schiff (PAS), which stains for mucin blue. Microscopic evaluation was performed at ×400 magnification, with 10 fields of view assessed per animal stomach. Images were captured using a digital camera attached to a light microscope. Mucin content was quantified following the method described by Medeiros et al. (2003), using Image Pro-Plus v6.0 software to determine the staining intensity. The presence of mucin is expressed as the number of pixels per selected microscopic field.

### Data analysis

Parameters, including stomach pH, ulcerative lesion index, mucus volume, and mucin content, were presented as mean values ± standard deviation, alongside minimum and maximum values, utilising the SAS statistical software package (SAS Institute Inc., NC), v8.0. The Kolmogorov-Smirnov test was used to assess normality, and Levene’s test was used to evaluate the homogeneity of variances. Subsequently, parametric assumptions were examined, and significant differences among the experimental groups were determined using one-way analysis of variance followed by Tukey’s post hoc test. A *P*-value below 0.05, denoted statistical significance, was set at *P < *0.05.

## RESULTS

HEG contains a diverse array of phenolic compounds with important antioxidant activities. The identified phenolic compounds include flavonoids and phenolic acids with specific subtypes, including chalcones, flavones, and flavonols. The extract demonstrated a robust capacity to scavenge the DPPH radical, yielding an IC_50_ value of 26.69 μg/ml, indicative of potent antioxidant properties.

**Figure 1:**
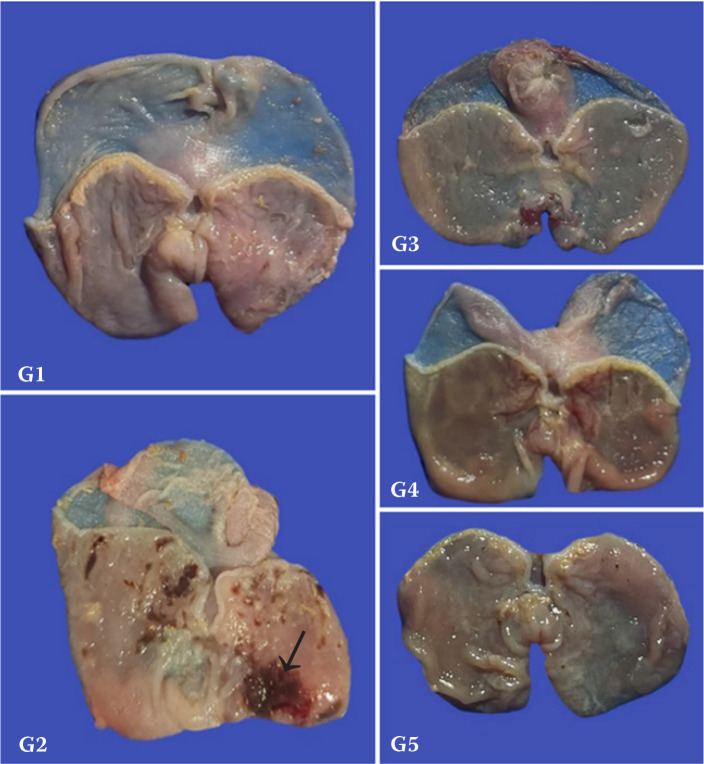
Macroscopic appearance of the stomach of rats in the experimental groups In G1, the mucosa showed no evidence of morphological changes; in G2, the mucosa showed oedema, a reddish colour, haemorrhage, and extensive ulcers (arrow); there were discrete areas of a reddish colour, small foci of erosion and ulceration of the mucosa in G3, G4, and G5 G1 = negative control, distilled water; G2 = positive control (ethanol 0.2 ml/animal); G3 = 250 mg/kg HEG; G4 = 500 mg/kg HEG; G5 = 1 000 mg/kg HEG

Macroscopic examination of the stomach revealed distinct findings in all the experimental groups (Figure 1). Animals in the negative control group (G1), administered distilled water, displayed normal morphological characteristics. In contrast, animals in the positive control group (G2) treated with ethanol exhibited severe mucosal damage, characterised by reddish, swollen, and haemorrhagic mucosa and extensive ulcers. The animals in groups G3, G4, and G5 displayed milder manifestations, including discrete reddish areas, focal erosion, and mucosal ulceration.

Significantly reduced ulcerative lesion indices were observed in animals from groups G3, G4, and G5 compared with those in group G2. Furthermore, a notable decrease in gastric secretion pH was noted in G2 animals compared to that in G1 animals. However, no significant differences were observed in gastric secretion pH between G3, G4, and G5 compared to G1. A significant reduction in the mucus volume collected from the stomach was also evident in G2 animals compared to G1 animals, with no significant differences observed in this parameter between G3, G4, and G5 animals compared to G1 animals (Table 1).

**Table 1:** Gastroprotective effect of the hydroethanolic extract of geopropolis (HEG) produced by *Melipona subnitida* D (Meliponinae, Apidae) against acute gastric injury caused by ethanol in rats

Treatments	Parameters evaluated			
	ulcerative lesion index	volume of mucus (ml)	stomach pH	containing mucin
G1	–	1.51 ± 0.65^A^	3.82 ± 0.93^A^	6 724 ± 4.32^A^
G2	27.43 ± 6.45^A^	0.68 ± 0.63^B^	2.15 ± 0.45^B^	827 ± 7.21^B^
G3	6.86 ± 4.30^B^	1.50 ± 0.48^A^	3.65 ± 0.82^A^	4 652 ± 4,58^A^
G4	3.29 ± 5.41^B^	1.64 ± 0.72^A^	3.80 ± 0.53^A^	5 021 ± 6.55^A^
G5	2.30 ± 5.87^B^	1.65 ± 0.58^A^	3.92 ± 0.72^A^	6 022 ± 8.02^A^

^A,B^Different letters in the mean values by column indicate statistical differences according to Tukey’s test (*P < *0.05)

G1 = negative control (distilled water); G2 = positive control (ethanol 0.2 ml/animal); G3 = 250 mg/kg HEG; G4 = 500 mg/kg HEG; G5 = 1 000 mg/kg HEG

**Figure 2:**
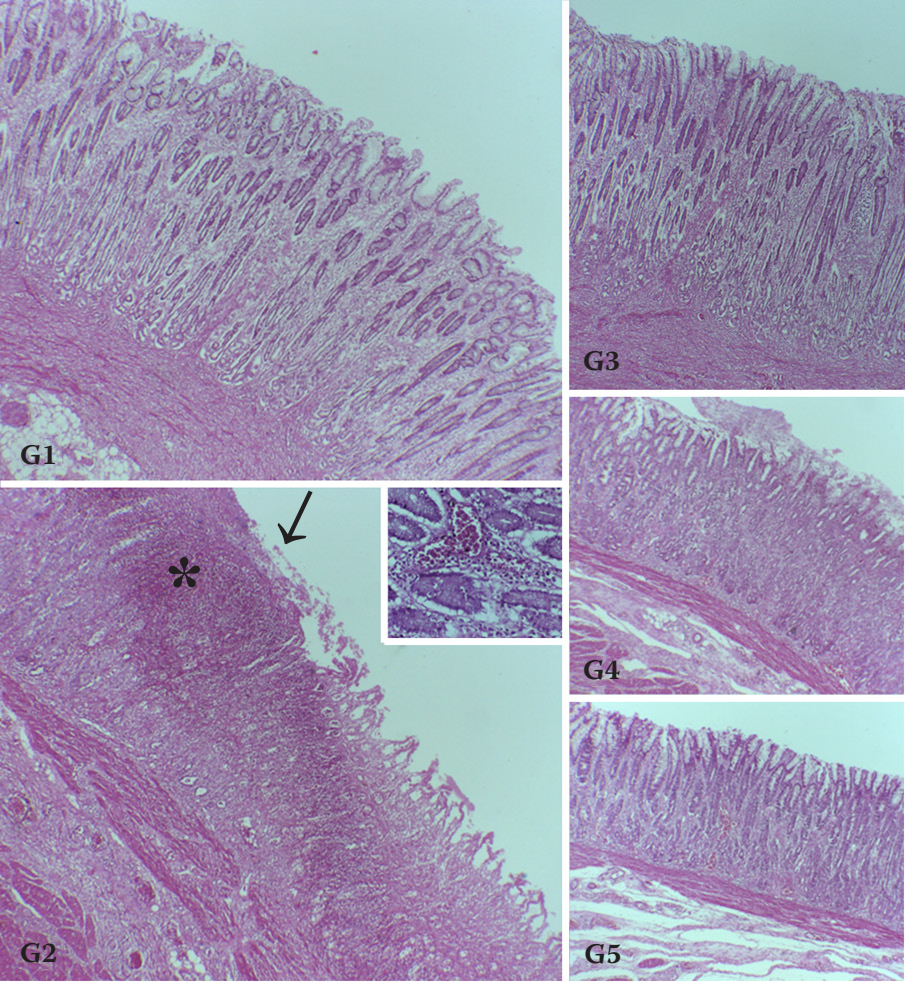
Histological photographs of the stomach sections of rats stained with haematoxylin and eosin in the experimental groups Normal histological architecture is observed in G1; total loss of the gastric mucosal epithelium (arrow), vascular congestion (inset), cell necrosis (*) and the presence of inflammatory cells in G2 and only slight congestion, oedema and focal detachment of the mucosal epithelium are observed in G3, G4 and G5. Scale bar = 50 μm G1 = negative control, distilled water; G2 = positive control (ethanol 0.2 ml/animal); G3 = 250 mg/kg HEG; G4 = 500 mg/kg HEG; G5 = 1 000 mg/kg HEG

Histological examination of stomach sections revealed distinct findings (Figure 2). Animals in group G1 exhibited no histological alterations, whereas those in group G2 displayed a total loss of gastric mucosal epithelium, vascular congestion, cell necrosis, marked haemorrhage, and infiltration of inflammatory cells, predominantly neutrophils. Animals in groups G3, G4, and G5 exhibited mild vascular congestion, focal haemorrhage, and focal detachment of the mucosal epithelium, with no evidence of a significant inflammatory response.

Histochemical analysis of mucin staining demonstrated a significant reduction in mucin concentration on the entire surface of the stomach mucosa in G2 animals compared to that in G1 animals. No significant differences in pixel values were observed among the animals in groups G3, G4, and G5, with values comparable to those in the G1 group.

## DISCUSSION

Gastric ulcers are prevalent ailments of the upper digestive tract, with a global incidence of 10% (Li et al. 2022). Consequently, numerous researchers are conducting studies about this condition, aiming to assess substances and develop therapeutic modalities either for their cure or, at the very least, for the improved patient’s quality of life. Various experimental models have been used to investigate gastric ailments. One such model involves inducing gastric ulcers through ethanol administration, which mimics several human-like characteristics of the condition and, thereby, serves as a platform for evaluating agents with potential gastroprotective effects (Arab et al. 2015).

The present study examined the effect of pre-treatment with HEG derived from *M. subnitida* on shielding the gastric mucosa against ethanol-induced damage in rats. These findings suggested that HEG has a gastroprotective effect. Notably, animals pre-treated with HEG exhibited a reduction in ethanol-induced lesions across all tested doses, with significant lesion diminishment observed even at the lowest dose (250 mg/kg).

It is plausible that the bioactive constituents of HEG contribute to the augmentation of endogenous mucosal protective mechanisms, potentially neutralising gastric acid and thereby elevating gastric pH while enhancing mucosal protection. The effect of propolis on gastric pH is significant, particularly in a subset of gastric diseases whose pathogenesis correlates with stomach acidity. Furthermore, the gastroprotective potential of the propolis extract was explored by assessing the gastric mucus volume. The results indicated a pronounced loss of mucus in the positive control group, which was solely administered ethanol (G2), in contrast with the notable increase in mucus production observed in animals treated with propolis extract.

When employing a histochemical technique involving PAS, which is specific for mucin staining, it was observed that pre-treatment with HEG resulted in the preservation of mucin content within the gastric mucus. This preservation was in contrast to the observed reduction in mucin levels among animals in the G2 group. The gastric epithelium secretes alkaline mucus, forming an adherent layer of viscoelastic mucous gel that serves as a protective physical barrier, inhibiting self-digestion of the mucosa by the acidic and enzymatic components present in the gastric juice (Flemstrom and Isenberg 2001).

Gastric mucus primarily consists of mucins, which are high-molecular-weight glycoproteins responsible for the texture and viscosity of the mucus (McGuckin et al. 2011).

Consequently, based on the data acquired in this study, it may be inferred that HEG exerts a protective effect on the gastric mucosa against the detrimental effects of ethanol by promoting an increase in mucus production and preserving mucin content.

These findings were consistent with those reported by Mendonca et al. (2020). The authors investigated the gastroprotective properties of hydroalcoholic extracts of red propolis (HERP) derived from *Apis mellifera* collected in Sergipe, Northeast Brazil. Using an ethanol-induced gastric ulcer model in rats treated orally with HERP at doses ranging from 50–500 mg/kg, the authors demonstrated the gastroprotective effect of HERP, as evidenced by a significant reduction (*P < *0.001) in the total area of gastric ulcers and an increase in gastric mucus production.

Ethanol directly damages gastric mucosal epithelial cells and induces oxidative stress and inflammation (Chen et al. 2021). The pathogenesis of ethanol-induced ulcers is closely associated with the deleterious effects of reactive oxygen species (ROS). Ethanol is metabolised by alcohol dehydrogenase (ADH) to acetaldehyde, a toxic metabolite that exacerbates oxidative stress and ROS formation (Yadav et al. 2013). ROS contributes to tissue damage by promoting lipid peroxidation, depleting intracellular glutathione (a natural antioxidant), elevating cytosolic calcium levels, and activating proteases, culminating in apoptosis (Muthuraman and Sood 2010; Liu et al. 2015).

The presence of phenolic compounds such as flavonoids, chalcones, flavones, flavonols, and phenolic acids in the HEG used in this study, along with its potent antioxidant activity, may account for its gastroprotective efficacy. Phenolic compounds, particularly flavonoids, are recognised as the key bioactive constituents of propolis produced by bees of the Meliponini tribe native to Brazil (Silva et al. 2016; Ferreira et al. 2017; Santos et al. 2017; Batista et al. 2020; Fontoura et al. 2020; Paiva et al. 2023). Flavonoids, a subgroup of phenolic compounds, possess therapeutic properties, such as antioxidant, anti-inflammatory, and wound-healing activities (Moraes et al. 2022). Phenolic compounds exhibit potent antioxidant properties by engaging in redox reactions and scavenging reactive oxygen species (Kataoka and Cardoso 2013). Furthermore, flavonoids exert gastroprotective effects by stimulating mucus and bicarbonate secretion (Gracioso et al. 2002).

Over the past decade, numerous studies have investigated the pharmacological effects of phenolic compounds on human health, particularly on the gastrointestinal tract. Phenolic compounds have various pharmacological effects, such as anti-secretory agents, cytoprotectants, and antioxidants. Several polyphenolic compounds exhibit antiulcer activity and substantial gastric protection. Consequently, phenolic compounds represent a promising therapeutic avenue for preventing or ameliorating gastric lesions induced by diverse ulcerogenic agents (Sumbul et al. 2011; Mokam et al. 2024).

The diversity of phenolic compounds and the potent antioxidant activity observed in the HEG used in this study indicate the high quality of the bee product. Although the gastroprotective efficacy of HEG, as demonstrated in this study, has not been thoroughly explored in scientific experiments, the data generated herein may contribute to validating the medicinal use of geopropolis. Thus, geopropolis may be a cost-effective alternative for preventing gastric lesions.

The obtained results demonstrate that the hydroethanolic extract of geopropolis produced by *M. subnitida* exhibits gastroprotective activity in the ethanol-induced ulcer model in Wistar rats. The extract effectively reduced the area of ulcerative lesions, mitigated the severity of both macroscopic and histological ethanol-induced changes, and stimulated increased mucus production, while maintaining mucin content and gastric pH levels. The gastroprotective efficacy of the extract was likely attributable to the presence of phenolic compounds and potent antioxidant activity.
